# Measurement of swept level distortion product otoacoustic emission growth functions at multiple frequencies simultaneously

**DOI:** 10.1121/10.0019578

**Published:** 2023-06-01

**Authors:** Courtney Coburn Glavin, Sumitrajit Dhar, Shawn S. Goodman

**Affiliations:** 1 Roxelyn and Richard Pepper Department of Communication Sciences and Disorders, Northwestern University, Evanston, Illinois 60208, USA; 2 Department of Communication Sciences and Disorders, University of Iowa, Iowa City, Iowa 52242, USA courtneyglavin@u.northwestern.edu, s-dhar@northwestern.edu, shawn-goodman@uiowa.edu

## Abstract

Otoacoustic emissions (OAEs) are low-level sounds generated by the inner ear that provide a non-invasive assessment of cochlear health. Advanced applications require recording OAEs across a wide range of frequencies and stimulus levels. Detailed here is a method for efficiently measuring distortion product otoacoustic emissions (DPOAEs) across an expansive stimulus space. Specifically, DPOAEs are recorded by sweeping the evoking stimuli in level across multiple frequencies simultaneously. This method generates DPOAE growth functions at multiple *f_2_* frequencies in several minutes. Results indicate the swept level method yields DPOAEs equivalent to those measured in a traditional (discrete stimulus) paradigm, but with several advantages.

## 1. Introduction

Otoacoustic emissions (OAEs) are commonly used in clinical applications as a screening tool to detect the presence or absence of hearing loss. However, advanced applications aim to provide a more comprehensive assessment of cochlear function. This may necessitate assessing OAEs over a wider range of stimulus conditions. For example, DPOAE growth functions—the pattern of DPOAE growth with increasing stimulus level at a given frequency—are sensitive to subclinical cochlear aging (Abdala *et al.*, 2021; Glavin *et al.*, 2021; Ortmann and Abdala, 2016). Additionally, cochlear insults, including aging, tend to first affect the highest frequencies of hearing (Bunch, 1929; Lee *et al.*, 2012; Northern *et al.*, 1972; Wiley *et al.*, 1998). This indicates the need to measure OAEs across a broad range of levels and frequencies to obtain a full picture of cochlear health and function.

In the most common conventional paradigm, DPOAE growth functions are measured by playing a pair of stimulus tones at adjacent frequencies at one level or level combination. Responses to the discrete stimuli are recorded until the signal of interest (OAE) can be extracted from the noise with confidence. This process is then repeated, as desired, using various combinations of stimuli to obtain information across level(s) and frequency range(s) of interest.

Discrete recording paradigms likely suffice when using OAEs as a screening tool (i.e., recording at only one level combination and a limited number of frequencies). However, inefficiencies may arise when attempting to record OAE growth functions (which necessitate measurement at many level combinations). First, depending on the number and range of discrete stimulus level(s) tested, the data may be too sparse to accurately capture the true nature of an individual's OAE growth. Various features of OAE growth functions, including threshold (Kummer *et al.*, 1998; Neely *et al.*, 2003), points of maximum strength or gain (Abdala *et al.*, 2022), and inflection points and/or slopes (Abdala *et al.*, 2022; Abdala and Kalluri, 2017; Gates *et al.*, 2002; Glavin *et al.*, 2021) have previously been reported. These extracted features have subsequently been related to cochlear pathology (Gates *et al.* 2002), psychophysical phenomena [e.g., loudness growth (Rasetshwane *et al.*, 2013)], and other demographic characteristics [e.g., age (Ortmann and Abdala, 2016)]. Independent growth functions of DPOAE components (distortion and reflection) have also been measured using brief pulsed stimuli (Zelle *et al.*, 2017a; Zelle *et al.*, 2017b). Capturing the true shape of the growth function is of particular concern given that the discrete measurement levels are typically selected *a priori*, and that there is significant variability in OAE growth between individuals—even among those who are similar demographically (Glavin *et al.* 2021).

Second, and relatedly, discrete measurement paradigms may compromise OAE growth function goodness of fit across individuals—particularly when using models with fewer parameters. In a previous analysis, we used a three-segment piecewise linear fitting to characterize DPOAE growth functions (Muggeo, 2003). While most growth functions could be fit with this technique (566/568), up to 10% of the generated fittings were poor [i.e., did not closely match the measured data (Glavin *et al.*, 2021)]. This was true even with densely sampled growth functions measured using a relatively small stimulus level step size (2 dB). In our experience, larger step sizes between discrete stimuli further limit the ability to accurately fit OAE growth functions with more easily interpretable models.

Here, we detail a method to continuously vary the level of tonal stimuli used to evoke DPOAEs and to present these swept level stimuli at multiple frequency combinations simultaneously. We verify the method by demonstrating that DPOAEs recorded using swept level stimuli are equivalent to those measured using a discrete stimulus (traditional) paradigm. The proposed swept level paradigm is an efficient method for measuring DPOAE growth, as it can yield near-continuous growth functions at multiple *f_2_* frequencies in several minutes.

## 2. Methods

### 2.1 Participants

Participants included six adults (F = 6; age range: 16–35 years) with self-reported normal hearing and audiometric thresholds ≤ 20 dB HL from 0.25 to 8 kHz bilaterally. Two participants self-identified their race as Asian, three self-identified as White, and one chose not to disclose. No participants self-reported their ethnicity as Hispanic or Latino. All participants consented to participate in this research, and all procedures were followed in accordance with and in approval by the Northwestern University IRB.

All DPOAEs were recorded from a randomly selected test ear of each participant. A screening protocol was run to assess the presence and robustness of DPOAEs in the participant's test ear from *f_2_* = 1–10 kHz at one stimulus level combination [65/55 dB forward pressure level (FPL); FPL is defined as a measure of the pressure level of the sum of all incident waves of the stimuli arriving at the eardrum, see Scheperle *et al.* (2008) for details] before growth functions were recorded. The screening protocol used a pair of stimulus tones, fixed in level and frequency ratio, continuously swept in frequency. Participants were included in the experiment only if their DPOAEs were sufficiently above the noise floor (≥3 dB) across all frequencies in this screening. If DPOAE growth functions were measured from a participant across multiple test sessions, the screener was repeated at each session to ensure adequate test-retest reliability.

### 2.2 Instrumentation and calibration

Audiometric thresholds from 0.25 to 8 kHz (at octave and inter-octave frequencies) were measured using Shoebox Audiometry (Bastianelli *et al.*, 2019) in a double-walled sound-treated booth. Shoebox Audiometry is automated and self-administered by the participant using an iPad and E-A-RTONE 3 A transducers coupled to the ear with foam tips.

DPOAEs were measured using an ER10X probe system (Interacoustics, Denmark; at the time of data collection: Etymotic, Elk Grove Village, IL). DPOAE signal generation and data collection were controlled using custom software (arlas, S.S.G.) written in matlab (Mathworks, Natick, MA, USA) on a PC running Windows 10. An RME Fireface UCX II 24-bit audio interface was used for A/D and D/A conversion.

DPOAE stimuli were calibrated using a forward pressure level (FPL) technique (Scheperle *et al.*, 2008). Recorded OAEs are reported in emitted pressure level (EPL) (Charaziak and Shera, 2017). Both procedures are described in detail elsewhere and are designed to minimize the effects of ear canal standing waves on OAEs.

### 2.3 DPOAE measurement

DPOAE growth functions were measured using both discrete and swept level stimuli in each participant's test ear at *f_2_* = 1, 4, 8, and/or 10 kHz. For a given recording condition, *f_1_* was set according to either *f_2_*/*f_1_* = 1.22 or *f_2_*/*f_1_* = 1.2, 1.2, 1.16, or 1.16 at *f_2_* = 1, 4, 8, and 10 kHz, respectively. These *f_2_*/*f_1_* ratios were selected to represent common clinical protocols (1.22 ratio) or to optimize DPOAE levels based on known mechanical properties of the cochlea [optimal ratios (Stiepan *et al.*, 2022)]. DPOAEs obtained using discrete and swept level stimuli were only compared across equivalent *f_2_*/*f_1_* ratio conditions.

To obtain a growth function, *L_1_* was fixed at 70 dB FPL. *L_2_* was varied from 10 to 70 dB FPL in 10 dB steps (discrete stimuli) or swept from 0 to 70 dB FPL at a rate of 10 dB/s (swept level stimuli). DPOAE growth functions measured using a fixed *L_1_* and varying *L_2_* most closely match analogous growth functions in basilar membrane displacement in guinea pig (Withnell and Yates, 1998). Additionally, this stimulus paradigm has been demonstrated to yield DPOAE growth functions that are sensitive to subclinical signs of cochlear aging (Glavin *et al.*, 2021).

Discrete stimuli were 1 s in duration. Each buffer consisted of three stimulus presentations and was repeated 32 times at each test condition. Swept level stimuli were 7 s in duration (before ramping and zero-padding) and were repeated 40 times per test condition. For discrete stimuli, test length for each level and frequency combination (e.g., 70/70 at *f_2_* = 1 kHz) was approximately 2–3 min. Thus, measurement of a coarse (10 dB step size) DPOAE growth function at a single *f_2_* frequency took approximately 15 min. In contrast, multiple DPOAE growth functions measured using swept level stimuli could be obtained in less than 4 min (when measuring at multiple *f_2_* frequencies simultaneously). That is, the growth functions measured using discrete level stimuli were obtained while presenting a single pair of tones during a given measurement window. In contrast, growth functions for swept level tones were obtained using multiple pairs of tones presented simultaneously at different frequencies. For example, the growth function for *f_2_* = 1 kHz was recorded in isolation as well as with growth functions at one or more test frequencies (*f_2_* = 4, 8, and 10 kHz). We acknowledge that parameter settings (e.g., stimulus length, number of repetitions) can be varied to impact overall test time. These parameters should be selected according to the application. This concept is further explored in Sec. 4. Therefore, the recording parameters used here should not suggest that the swept level method will necessarily be faster than the discrete stimulus method by a factor of 3 to 4.

### 2.4 DPOAE analysis

Data recorded using discrete stimuli were analyzed using a fast Fourier transform (FFT). Data recorded using the swept level method were analyzed using sliding time analysis windows (200 ms in length, ∼17 ms of overlap between windows) with a weighted least squares fitting (WLSF) procedure. The ordinary LSF procedure for estimating the 2*f_1_*-*f_2_* total DPOAE is described in detail in previous publications (Long *et al.*, 2008; Long and Talmadge, 1997; Talmadge *et al.*, 1999). In short, the LSF procedure scales a model DPOAE response to minimize the sum of squares error between the model and measured pressure. To fit data collected with swept level stimuli, we used sinusoidal basis functions of unit amplitude multiplied by a Blackman window. For swept stimuli, the WLSF procedure is preferable because it allows noisy samples to be removed from the analysis without discarding the entire sweep in which they occurred. Weighting values of zero were assigned to noisy time samples, identified by considering the variance across repeated sweeps. For each sweep, the WLSF model was used to obtain coefficients for the cosine- and sine-phase portions of the model DPOAE. The coefficients were combined into complex form (similar to complex Fourier coefficients). The DPOAE signal was then calculated as the magnitude of the mean of the complex coefficients across repeated sweeps (i.e., across stimulus repetitions). The noise floor (for both discrete and swept level data) was calculated as the standard error of the mean across repeated sweeps.

## 3. Results

### 3.1 Discrete vs swept level DPOAE estimates

**Fig. 1.**
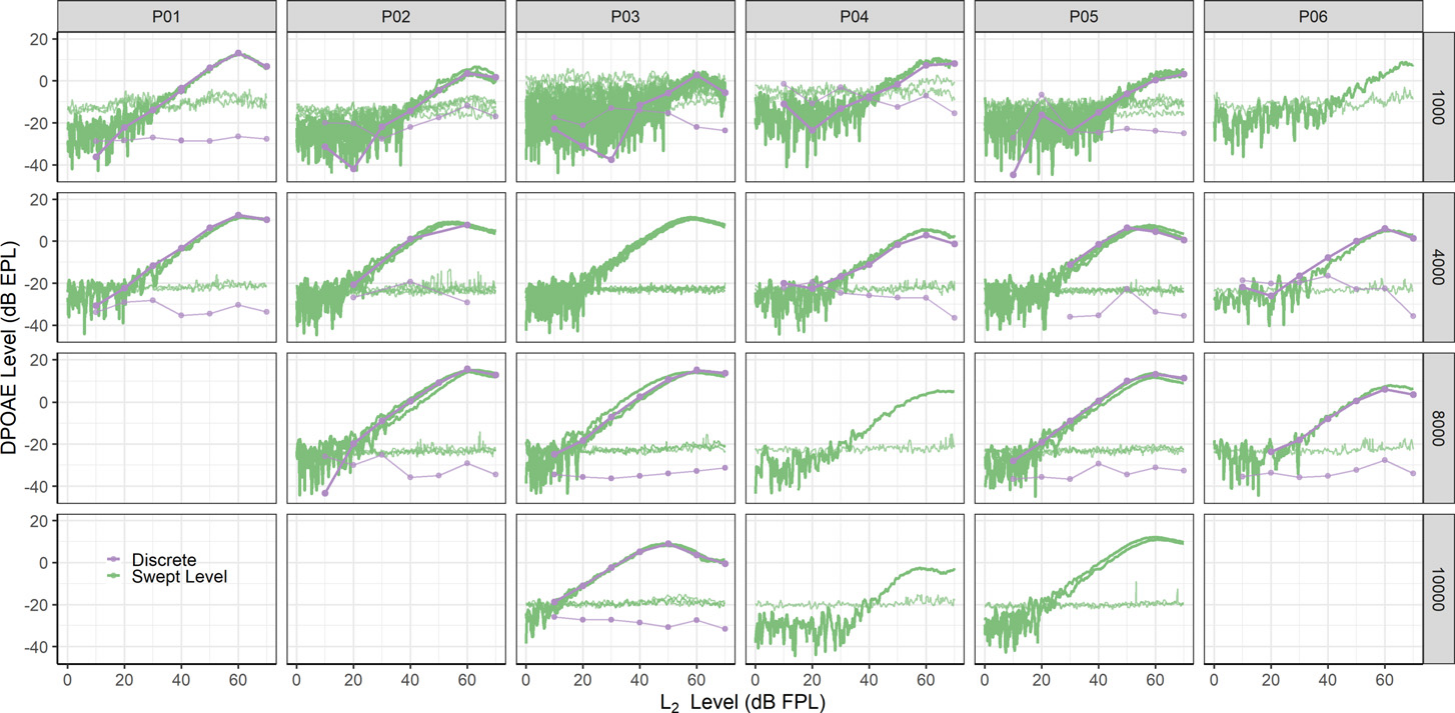
Individual DPOAE growth functions measured with discrete (purple) and swept level (green) stimuli yield equivalent results at four *f_2_* frequencies across participants. Associated noise floors for each stimulus paradigm are also shown. Blank panels indicate that a participant did not have measured data at that particular *f_2_* frequency. Panels missing discrete data indicate that discrete data could not be compared to swept level data for that participant/frequency combination because discrete data were not measured with an optimal *f_2_/f_1_* ratio.

**Fig. 2.**
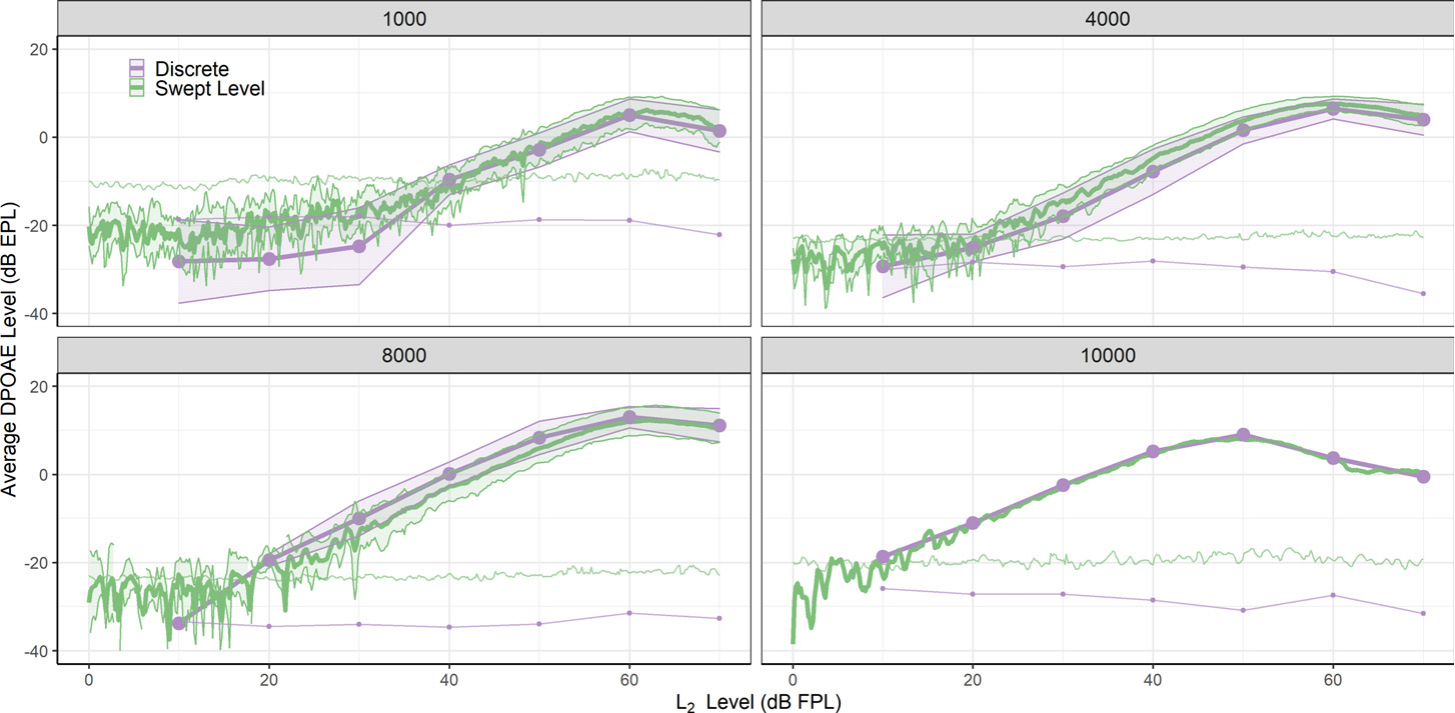
Average DPOAE growth functions measured with discrete (purple) and swept level (green) stimuli yield equivalent results at four *f_2_* frequencies. Associated noise floors for each stimulus paradigm are also shown. The shaded regions around each DPOAE growth curve represent twice the standard error of the mean. One growth function from each stimulus condition for each participant was chosen for averaging. Note that *f_2_* = 10 kHz has data from only one participant.

DPOAE growth functions measured using swept level stimuli closely resembled those measured using discrete tones. This is highlighted across individual participants in Fig. 1 and on average in Fig. 2. Figure 1 displays DPOAE growth functions from each participant measured using both paradigms (discrete and swept level) at each test frequency. Some participants had multiple growth functions measured within a given stimulus/frequency condition (all of which are shown), while others did not have a growth function for a given condition (indicated by blank panels). Only data with matched *f_2_/f_1_* ratios between stimulus conditions are shown. For each participant, both stimulus conditions yielded similar DPOAE estimates. While there are noticeable differences in noise floors between stimulus conditions, these are primarily due to differences in averaging time. Specifically, the noise floors estimated from the discrete stimulus condition tend to be lower because of longer averaging times. This is explored further in Sec. 4.

Figure 2 displays average DPOAE growth functions obtained with both paradigms at each *f_2_* frequency. To compare average differences between conditions, one DPOAE growth function obtained using each measurement paradigm (discrete and swept) was selected from each participant per *f_2_* frequency. In the cases where participants had multiple discrete or swept growth functions at a given *f_2_* frequency, one from each condition was selected randomly. In cases where participants did not have a growth function for either or both conditions at a given *f_2_* frequency, they were excluded from the average calculation at that *f_2_* frequency. Only growth functions with matched *f_2_/f_1_* ratios within a given participant between stimulus conditions were used to compare average differences. Prior to averaging, growth function data from each participant were cleaned by removing DPOAE data points that were less than 3 dB above the noise floor. Note that data are available from only one participant for *f_2_* = 10 kHz, primarily because all other participants had swept data collected with *f_2_/f_1_* = 1.16 while discrete data were collected with *f_2_/f_1_* = 1.22. This resulted in differences between growth functions that can be attributed to mechanical properties of the cochlea (Stiepan *et al.*, 2022) rather than measurement paradigm (not shown). Therefore, those growth functions were excluded from analysis here.

**Fig. 3.**
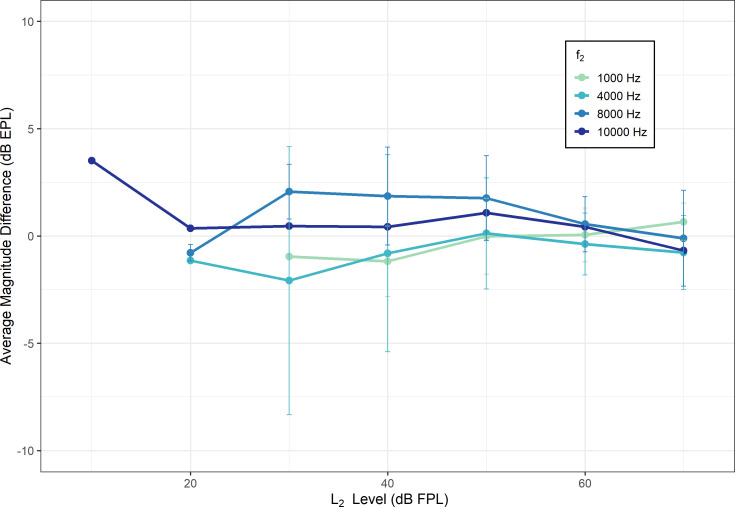
Average magnitude differences between DPOAE growth functions measured discretely vs those measured with swept level stimuli at each *f_2_* frequency (difference = discrete − swept). DPOAE data for each participant at a given level were included in the average calculation if they met an SNR criterion of >6 dB in both measurement paradigms (discrete and swept). A positive value indicates that the discretely measured DPOAE was higher in level. Error bars indicate ±1 standard deviation of the mean. Points without error bars indicate that data from only one participant are shown. Average differences between the two stimulus conditions are <5 dB at all frequencies.

Average differences between DPOAEs obtained with the two measurement paradigms (discrete vs swept) were within ±5 dB across all test frequencies, as shown in Fig. 3. Therefore, differences between stimulus conditions are consistent with typical test-retest reliability expected for OAE measurement at all *f*_2_ frequencies tested (Wagner *et al.*, 2008). To be included in the average difference calculation, DPOAE data from a given participant at a given level were required to have an SNR > 6 dB in both measurement paradigms. Maximal differences between stimulus conditions—particularly those greater than 5 dB—tended to occur at lower *L_2_* levels (*L_2_* ≲ 30 dB FPL). This is where the signal-to-noise ratio (SNR) of measured DPOAEs tended to be the smallest for both stimulus types before cleaning the data for SNR. Therefore, it is expected that differences between conditions are greatest here, as test-retest reliability is likely to be worse when SNR is poorer. Notably, variability between conditions was not higher near *f_2_* frequencies typically impacted by standing waves (e.g., *f_2_* = 4 and 10 kHz), reaffirming the advantages of FPL and EPL calibration techniques.

### 3.2 Repeatability of swept level DPOAEs

**Fig. 4.**
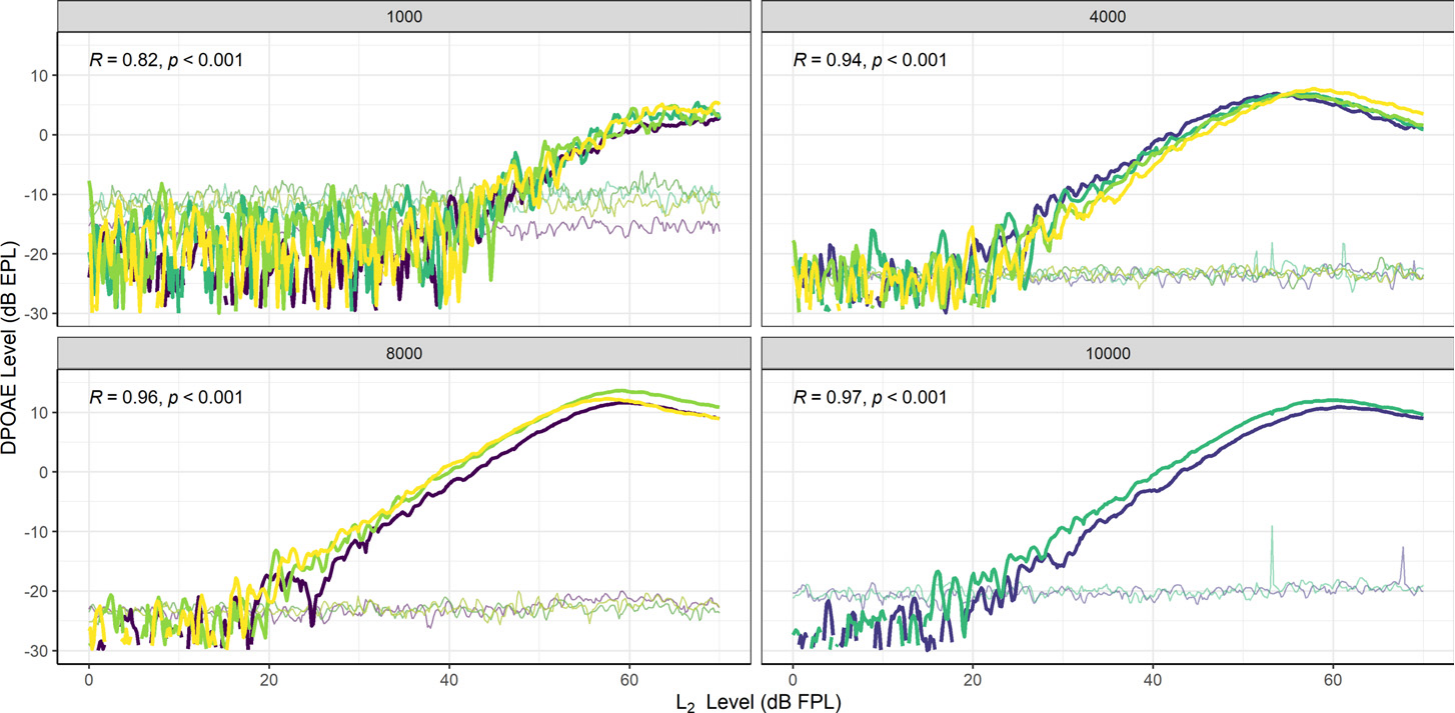
Repeated DPOAE growth functions and estimated noise floors from one representative participant measured using swept level stimuli at four *f_2_* frequencies. The color of each line indicates the run number and therefore shows which *f_2_* frequencies were measured simultaneously (e.g., the yellow lines in the 1, 4, and 8 kHz panels indicate that data from these three *f_2_* frequencies were measured together). These growth functions were obtained across probe insertions and test sessions. Results indicate high repeatability, as indicated by the Pearson correlation coefficient shown in each panel.

To explore the repeatability of DPOAEs obtained using swept level stimuli, swept growth functions were repeated across sessions in a subset of participants (n = 2). This subset of participants was selected for follow-up measures based on their availability to return for additional testing. In these participants, swept level DPOAEs were measured two or more times at a given *f_2_* frequency. Analysis of repeated swept level DPOAEs for one representative participant are reported here and are shown in Fig. 4. The data displayed from this participant includes measures across various probe insertions and test sessions. It also includes DPOAE growth functions measured with various simultaneously presented *f_2_* stimulus combinations. Pearson correlation coefficients were calculated at each frequency for this participant and indicated strong associations between sweeps—and therefore high test-retest reliability—at all frequencies: 1 kHz [*r*(407) = 0.82, *p* < 0.001], 4 kHz [*r*(407) = 0.94, *p* < 0.001], 8 kHz [*r*(407) = 0.96, *p* < 0.001], and 10 kHz [*r*(407) = 0.97, *p* < 0.001].

As previously stated, we also measured swept level data with either two, three, or four *f_2_* and *f_1_* frequency combinations presented simultaneously. Thus, for example, the frequency condition *f_2_* = 1 kHz may have been measured simultaneously with 1–2 other frequency conditions (e.g., *f_2_* = 1 and 8 kHz, or *f_2_* = 1, 4, and 8 kHz, or *f_2_* = 1, 4, 8, and 10 kHz). This was done as a proof of concept to verify that multiple frequency combinations tested simultaneously would yield equivalent results, and therefore could reduce recording time even further than a single swept level stimulus pair alone. All combinations of stimuli yielded equivalent results except when *f_2_* = 8 and 10 kHz were presented simultaneously. In this condition, the DPOAE growth function at *f_2_* = 8 kHz was reduced, indicative of two-tone suppression (not shown).

## 4. Discussion

Since the discovery of OAEs by David Kemp in 1978 (Kemp, 1978), decades of research has highlighted their clinical potential. In particular, recent work has shown the potential of using OAEs to detect subclinical aging (Abdala *et al.*, 2021; Glavin *et al.*, 2021; Ortmann and Abdala, 2016), to differentiate between cochlear pathologies (Gates *et al.*, 2002; Ueberfuhr *et al.*, 2016), and to predict behavioral thresholds (Kummer *et al.*, 1998; Neely *et al.*, 2003). However, these demonstrations in the laboratory have not translated to clinical practice, where OAEs continue to be used primarily as a screening tool to detect the presence or absence of hearing loss. In part, this is because using OAEs for more advanced applications requires increasing the stimulus space over which they are measured. There is a growing body of literature on more efficient measurement of OAEs [e.g., Abdala *et al.* (2015), Kalluri and Shera (2013), Long *et al.* (2008), and Neely *et al.* (2005)]. Much of this work has led to significant time-savings by using stimuli swept in frequency. Here, we validate the use of stimuli swept in level presented simultaneously at multiple *f_2_* frequencies for the efficient recording of DPOAE growth functions.

Our results show that DPOAEs measured using swept level stimuli are equivalent to those measured using traditional discrete stimuli, and that DPOAEs obtained using this method are highly repeatable. Thus, using swept level stimuli to measure DPOAE growth functions is both a valid and reliable approach. This approach may be particularly useful when attempting to efficiently determine the overall shape of an individual's growth function(s), which can vary widely across ears and may not be fully captured when sampling a growth function using discrete levels that are selected without prior knowledge of an individual's OAE growth. Additionally, a nearly continuous growth function may have the added advantage of improving the goodness of fit of *post hoc* models used to characterize it, especially when fitting a model with fewer parameters.

Slight discrepancies between the stimulus conditions were noted in Sec. 3, including a systematic trend of discretely measured noise floors being lower than noise floors estimated from swept level stimuli. This is due to differences in our measurement techniques between stimulus conditions. Specifically, discrete stimuli were 1 s in duration and repeated 96 times at a given *L_1_*/*L_2_* combination. This is equivalent to 96 s of stimulus presentation (and therefore averaging) at a given discrete point along the function. In contrast, the swept level stimuli varied from 0 to 70 dB FPL at a rate of 10 dB/s and repeated 40 times. Thus, while the duration of one stimulus was longer (7 s), it covered a much broader range of *L_2_* levels. It was therefore equivalent to ∼0.68 s (0.017 s * 40 repetitions) of stimulus presentation/averaging at a given discrete point along the growth function. Increased averaging is expected to decrease the estimated noise floor. Since the difference between estimated DPOAEs between the two conditions is within the range of expected test-retest reliability of OAEs (Wagner *et al.*, 2008) and noise floor differences could be further minimized by changing the recording parameters of swept stimuli (i.e., by changing the number of repetitions, reducing the sweep rate, and/or reducing the range of levels), this is not an underlying problem with swept level stimuli. These recording parameters can and should be varied depending on the application. For example, if estimating DPOAE growth function thresholds is a priority, averaging should be increased—at least at stimulus levels near the expected threshold.

A potential limitation of using swept level stimuli is the inability to separate DPOAE components using common approaches such as time-windowing. Notably, most of our growth functions measured with swept level stimuli are relatively smooth across levels; this lack of fine structure implies that the distortion component may be mostly dominant, at least in the ears tested here. This could be due, in part, to our choice of holding *L_1_* at a constant 70 dB FPL thereby limiting the phase variation between DPOAEs generated by adjacent generators. This limitation can be overcome by using pulsed stimulus tones that allow temporal separation of DPOAE components and therefore independent growth functions for DPOAE components (Zelle *et al.*, 2017a).

In addition to the gains of using swept level stimuli, we also demonstrate the feasibility of simultaneous stimulation with multiple frequency pairs thereby obtaining several growth functions in a brief period. Practically, our preliminary work suggests that *f_2_* frequencies should be spaced appropriately far apart so as to not lead to tone-on-tone suppression when recording multiple growth functions simultaneously. As noted in Sec. 3, the DPOAE growth function(s) measured at *f_2_*** **=** **8 kHz were lower when measured simultaneously with the *f_2_*** **=** **10 kHz stimulus combination. This is likely because when *f_2_*** **=** **10 kHz, *f_1_* =** **8.62 kHz with our stimulus parameters (*f_2_*/*f_1_* =** **1.16). Thus, *f_1_* was close enough and high enough in level (*L_1_* =** **70 dB FPL) to suppress *f_2_*** **=** **8 kHz. While additional testing is required to determine optimal stimulus spacing, we suggest that setting *f_2_* frequencies a minimum of one octave apart should be a sufficient starting point. If measuring across the frequency range of human hearing, this would allow for up to six *L_2_* sweeps (e.g., *f_2_*** **=** **0.5, 1, 2, 4, 8, and 16 kHz) simultaneously. Future work should determine if stimulus spacing could be reduced further (e.g., half of an octave apart).

Additionally, while this work focuses on recording DPOAE growth functions using swept level stimuli, this measurement paradigm could be extended. For example, DPOAE growth functions could also be obtained by using multiple swept level stimuli presented simultaneously to co-vary in level [i.e., the “Scissors” paradigm (Kummer *et al.*, 1998)]. Swept level stimuli could also be used to obtain other types of OAE growth functions, including stimulus frequency OAEs (SFOAEs).

## 5. Conclusion

DPOAE growth functions measured with swept level stimuli at multiple *f_2_* frequencies simultaneously are equivalent to those measured using a traditional stimulus (discrete) paradigm. Use of this measurement technique may clear the way for more advanced OAE applications to be adopted both in clinical and research settings.
